# The composition of T-cell subsets are altered in the burn wound early after injury

**DOI:** 10.1371/journal.pone.0179015

**Published:** 2017-06-02

**Authors:** Meenakshi Rani, Martin G. Schwacha

**Affiliations:** Department of Surgery, The University of Texas Health Science Center at San Antonio, San Antonio, Texas, United States of America; Georgia Regents University Cancer Center, UNITED STATES

## Abstract

**Background:**

Burn-induced inflammation leads to impaired immune responses resulting in increased morbidity and mortality. T-cells are central in the immune response and circulating CD4 and CD8 T-cells have been used to evaluate immune status; however, the role of these T-cell subsets in the burn wound is unknown.

**Methods:**

Male C57BL/6 mice were subjected to a major 3^rd^ degree scald burn or sham treatment. Twenty-four hours later, full thickness skin samples from sham mice and the burn wounds were collected and single cells were isolated and analyzed for αβ TCR, γδ TCR, CD3, CD4, CD8 and CD69 expressions by flow cytometry.

**Results:**

The burn wound contained significantly greater numbers of T-cells than skin from sham mice, due to a profound infiltration of αβ T-cells. These infiltrating αβ T-cells were primarily suppressor T-cells with a CD8^+^ or CD8^-^CD4^-^ phenotype. The 15-fold increase in CD8^+^ αβ T-cells caused a decrease in the CD4:CD8 ratio from 0.7 in sham skin to 0.3 in the burn wound. In contrast, the majority of the γδ T-cells in sham skin were CD4^-^CD8^-^, which decreased 9-fold in the burn wound. CD69 expression was suppressed on burn wound αβ T-cells, but increased on γδ T-cells in the burn wound.

**Conclusions:**

The infiltrating burn wound αβ T-cells likely act to quell inflammation. In contrast wound γδ T-cells were activated with elevated CD4 and CD69 expression. Thus, these two distinct T-cell subsets likely differentially regulate the burn wound inflammatory response.

## Introduction

Major burn causes immune dysfunction that may contribute to wound healing complications and poor outcomes [1–3]. Various immune cells (i.e., neutrophils, macrophages and T-cells) play unique roles in orchestrating the immune and inflammatory responses and thereby regulate wound healing. Characterization of T-cell subsets and their activation status may provide further insight into the basis of these immunological changes and burn wound healing.

Various studies suggest that T-cells exert an important role in skin healing [4, 5]. T-cells are the most predominate lymphocyte subset in human skin wounds, and they migrate into wounds and peak during the late proliferative and early remodeling phases [6, 7]. Our previous findings have shown the role of T-cells in healing of the burn wound. These studies have demonstrated that γδ T-cells are essential in the wound healing response [8], associated with the development of a Th-2 and Th-17 response [9] and are activated and responsible for the infiltration of an αβ T-cell population [10].

Previous studies suggest that CD4^+^ and CD8^+^ T-cell subsets and CD4:CD8 ratio play a central role in the induction of efficient immune responses against different diseases such as human immunodeficiency virus (HIV), tuberculosis, and cancer [11–14]. Previous studies have examined the CD4:CD8 ratio and the characterization of these cells in the circulation [15, 16], as well as in the lymph nodes and scar tissues [4, 17]. With regard to the burn wound, little is known about CD4 and CD8 T cell subsets.

## Materials and methods

### Animals

C57BL/6 male mice (12–14 week old; Jackson Laboratories, Bar Harbor, ME, USA) were used in the experiments described herein. The animals were allowed to acclimatize for at least one week prior to experimentation and they were kept in ventilated cages under specific pathogen-free conditions. Mice were randomly assigned into either sham or burn group. All animal protocols were approved by the Institutional Animal Care and Use Committee (IACUC) of the University of Texas Health Science Center at San Antonio, and all procedures were performed in accordance with the National Institutes of Health guidelines for the care and handling of laboratory animals.

### Burn injury procedure

Mice received a scald burn as described previously [18]. Prior to the procedure the mice were anesthetized with ketamine/xylazine (i.p.). The dorsal surface was shaved and the anesthetized animal was placed in a custom insulated mold exposing 12.5% of their total body surface area (TBSA). The mold was immersed in 70°C water for 10 sec, producing a full-thickness burn [18]. The burn procedure was repeated on the both sides resulting in a 25% TBSA burn. The mice were then resuscitated with 1 ml of Ringer's lactate solution (i.p). Sham treatment consisted of anesthesia and resuscitation only.

### Skin tissue collection and single cell isolation

Twenty-four hours after burn or sham procedure, skin samples were collected and wet weight was measured. Normal non-injured skin was collected from sham, and injured skin from the burn site was collected from burn mice. Skin samples from the burn site included injured skin and the wound margin. The burn-injured skin was excised, down to the level of the musculofascia, including the submucosal layer by sharp dissection. The skin tissue was used to isolate single cells for flow cytometry.

Full thickness skin tissues were collected and processed to isolate single cells as previously described elsewhere [10]. Briefly, skin tissues were collected and minced into small pieces of approximately 2–3 mm in size and put into dispase II (0.05%, Roche) medium for overnight digestion at 4°C. The next day, skin samples were further minced into smaller pieces and then digested using trypsin-GNK (0.3%, Glucose/dextrose, NaCl and KCl buffer, Sigma) for 30 min at 37°C in a shaker waterbath. Heat inactivated Fetal Bovine Serum (FBS, GIBCO) was added to stop the reaction. The dissociated cells were sieved through a 100 μm mesh. The cell suspension was centrifuged and the cell pellet was resuspended in RPMI containing 10% heat-inactivated FBS (GIBCO), 50 μM of 2-Mercaptoethanol (Sigma-Aldrich), 2 mM of L-glutamine (GIBCO), 1 mM of sodium pyruvate (GIBCO), 100 μM Non-essential amino acids (GIBCO), 50 U/ml penicillin and 50 μg/ml streptomycin (GIBCO) supplemented with 10 U/ml murine recombinant IL-2 (BD Biosciences). Cells were counted and cultured in a 12-well plate at a density of 1x10^6^/ml overnight. The cells were collected after passing through a 70 μm mesh and used for flow cytometry the following day.

### T-cell phenotyping by flow cytometry

The isolated skin cells were washed in staining buffer (PBS with 0.2% BSA and 0.09% NaN_3_) and treated with Fc-blocking antibody (anti-CD16/CD32, BD Biosciences) for 15 min. The cells were then stained with the following directly conjugated antibodies: anti- CD3 (PE or APC-Cy7) in combination with anti-β TCR (PerCPCy5.5), anti-δ TCR (FITC), anti-CD4 (PECy7), anti-CD8 (APC-Cy7) and anti-CD69 (PE-Cy7). After 30 min of incubation on ice, the cells were washed and resuspended in staining buffer. Appropriate isotype controls were used for all staining. All data were acquired using a LSRII (BD Biosciences) and analyzed using FlowJo (Tree Star) software. A minimum of 50,000 events was collected and live cells were gated according to forward- and side-scatter properties. Absolute cell count was determined as % positive cells obtained as follows:

flow cytometry % x total number of cells per gm of wet weight100

CD4^+^ and CD8^+^ subsets were gated on total T-cells from sham and burn mice and cell count for these subsets was determined as % positive cells (CD4^+^ or CD8^+^ single positive cells). The CD4^+^ and CD8^+^ cell counts (as determined above) were used to determine the CD4:CD8 ratio of gated T-cells from sham and burn mice.

### Statistical analysis

Data are expressed as mean ± SEM. Comparisons were analyzed using ANOVA and student’s t-test was used for comparisons between two groups. A *p*-value of < 0.05 was considered to be statistically significant for all analyses.

## Results

### Increased numbers of T-cells are present in the burn wound

**Fig 1:**
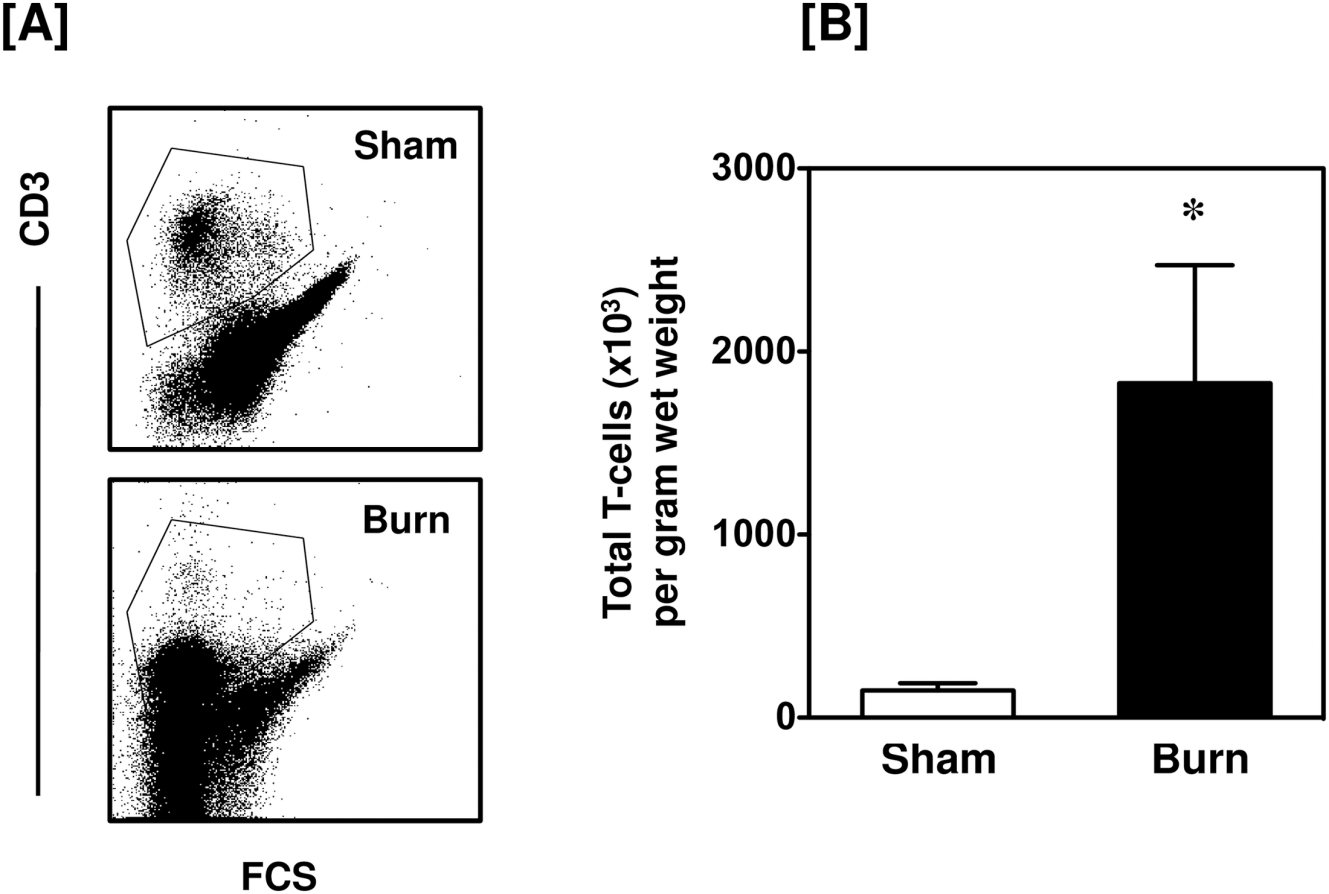
Total T-cells. After 24 h of sham or burn procedure, skin cells were studied for total T-cells using flow cytometry. [A] Gating strategy and representative dot plots of CD3^+^ T-cells from sham (upper panel) and burn mice (lower panel). [B] The number of total T-cells/g wet weight of the skin tissue from sham and burn mice. Data are mean ± SEM for 5–9 mice/group; *p < 0.05 vs. sham.

**Fig 2:**
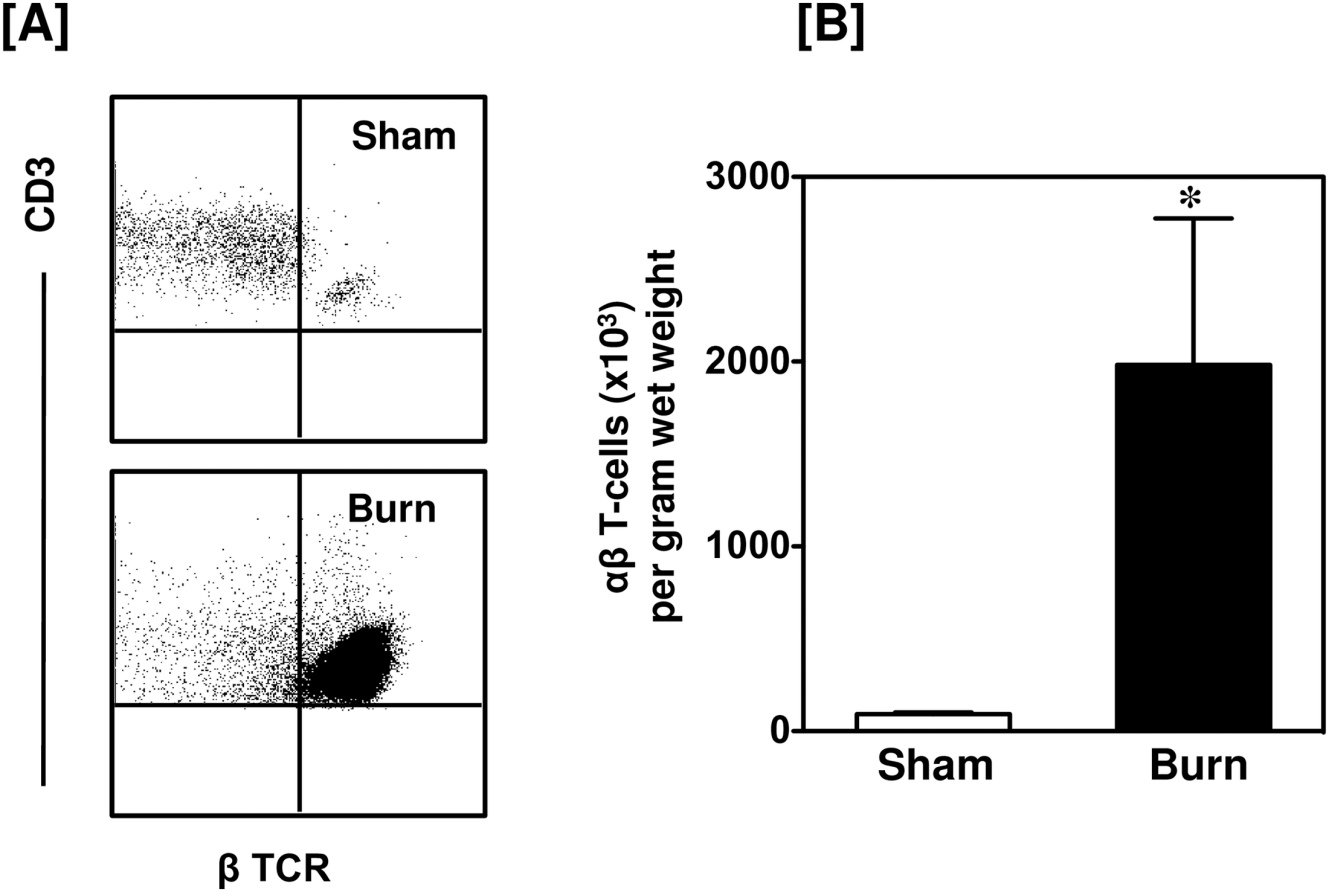
Total αβ T-cells. After 24 h of sham or burn procedure, skin cells were studied for αβ T-cells using flow cytometry. [A] Representative dot plots of β TCR^+^ αβ T-cells from sham (upper panel) and burn mice (lower panel). The αβ T-cells are gated from the total T-cell population. [B] The number of αβ T-cells/g wet weight of the skin tissue from sham and burn mice. Data are mean ± SEM for 5–9 mice/group; *p < 0.05 vs. sham.

**Fig 3:**
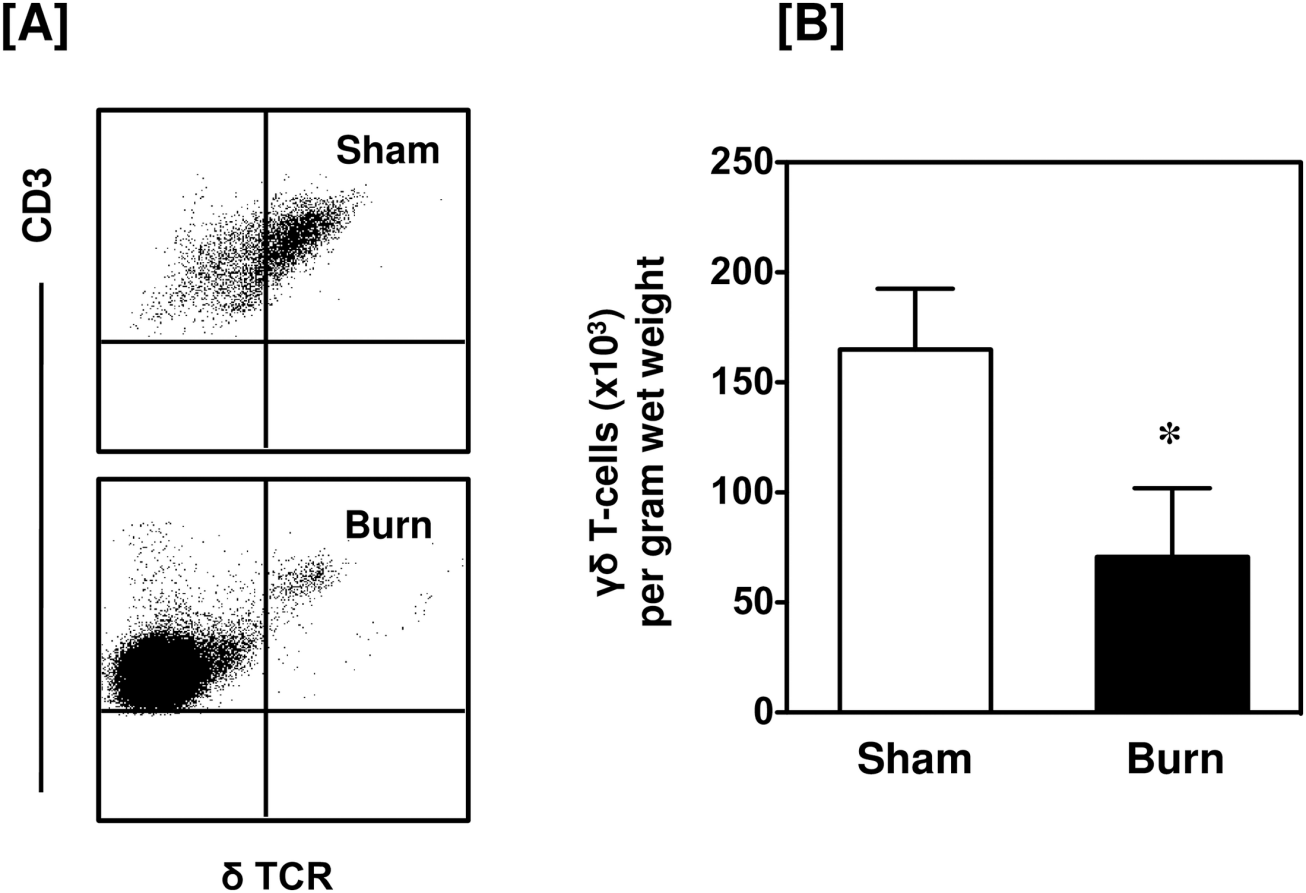
Total γδ T-cells. After 24 h of sham or burn procedure, skin cells were studied for γδ T-cells using flow cytometry. [A] Representative dot plots of δ TCR^+^ γδ T-cells from sham (upper panel) and burn mice (lower panel). The γδ T-cells are gated from the total T-cell population. [B] The number of γδ T-cells/g wet weight of the skin tissue from sham and burn mice. Data are mean ± SEM for 5–9 mice/group; *p < 0.05 vs. sham.

Burn injury induced a marked influx of CD3^+^ T-cells into the wound site (Fig 1). A greater than 20-fold increase in the number of T-cells in the injured skin was observed as compared with uninjured skin from sham mice. Under normal conditions (sham skin) only 6.8% of the skin cells were CD3^+^, whereas 24% of the skin cells were CD3^+^ in the burn wound. Further analysis of the CD3^+^ cells revealed that the influxing T-cells were positive for αβ TCR (Fig 2). In contrast, in sham skin γδ T-cells were the predominant T-cell population and their numbers were slightly, but significantly, reduced in the burn wound. (Fig 3).

### After injury CD4 and CD8 expression differs between αβ and γδ T-cells

**Fig 4:**
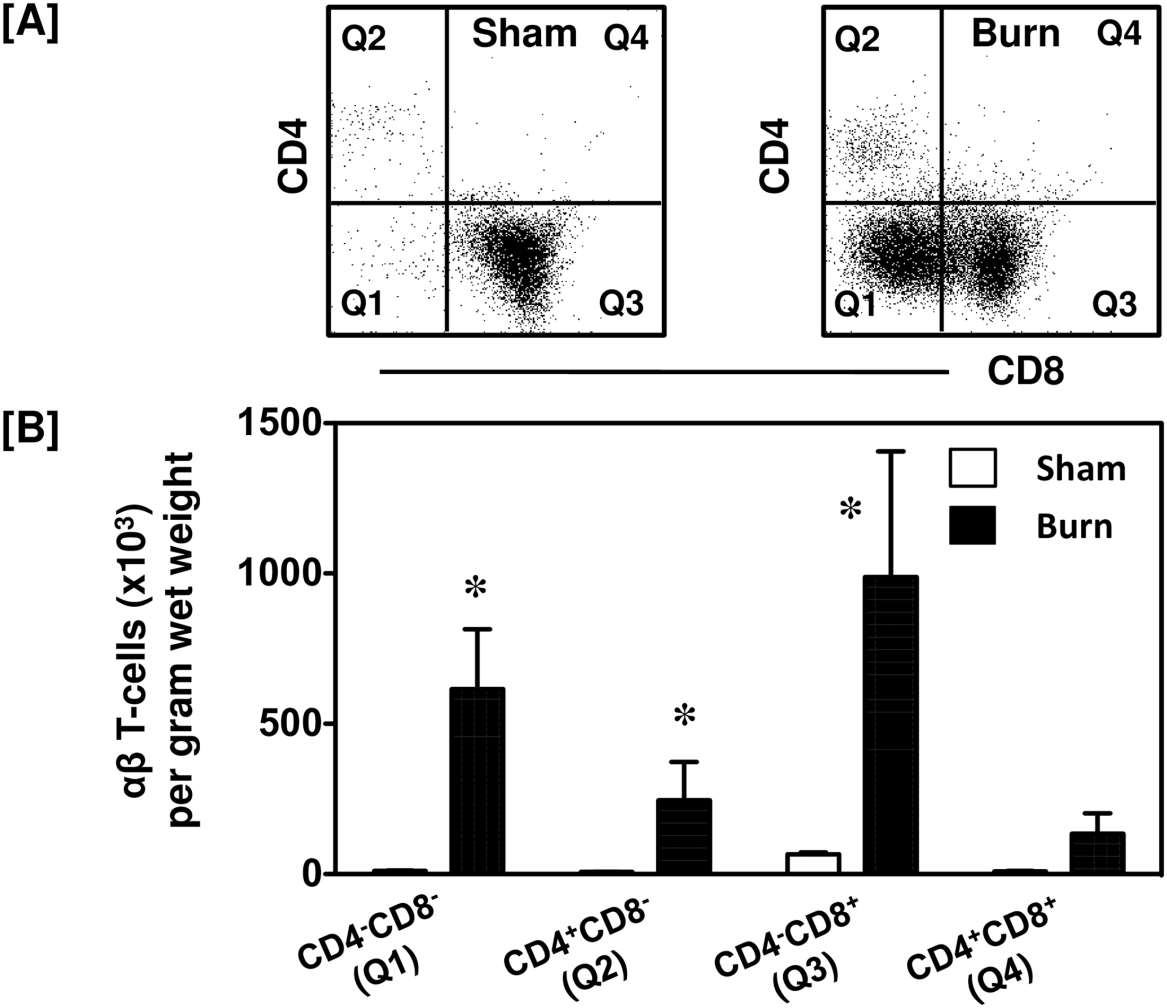
CD4 and CD8 populations of αβ T-cells. After 24 h of sham or burn procedure, skin cells were studied to characterize CD4 and CD8 populations of αβ T-cells using flow cytometry. [A] Gating strategy demonstrating CD4^-^CD8^-^ (Q1), CD4^+^CD8^-^ (Q2), CD4^-^CD8^+^ (Q3) and CD4^+^CD8^+^ (Q4) αβ T-cells from sham and burn mice. [B] Graphical representation of CD4 and CD8 populations of αβ T-cells. Data are mean ± SEM for 5–9 mice/group; *p < 0.05 vs. respective sham.

Further characterization of the αβ T-cells in the skin revealed that while they were a limited population in the sham skin, the αβ T-cells present were mainly CD8^+^ (71%; Fig 4A). After burn, there was a huge infiltration of αβ T-cells into the wound site (Fig 4). Forty-eight percent of the burn wound αβ T-cells were CD8^+^ T-cells, whereas approximately 30% of αβ T-cells belonged to CD8^-^CD4^-^ population (Fig 4A). When compared to sham mice the actual numbers of CD4^-^CD8^-^, CD4^+^CD8^-^ and CD4^-^CD8^+^ αβ T-cells were significantly increased in the burn wound (Fig 4B).

**Fig 5:**
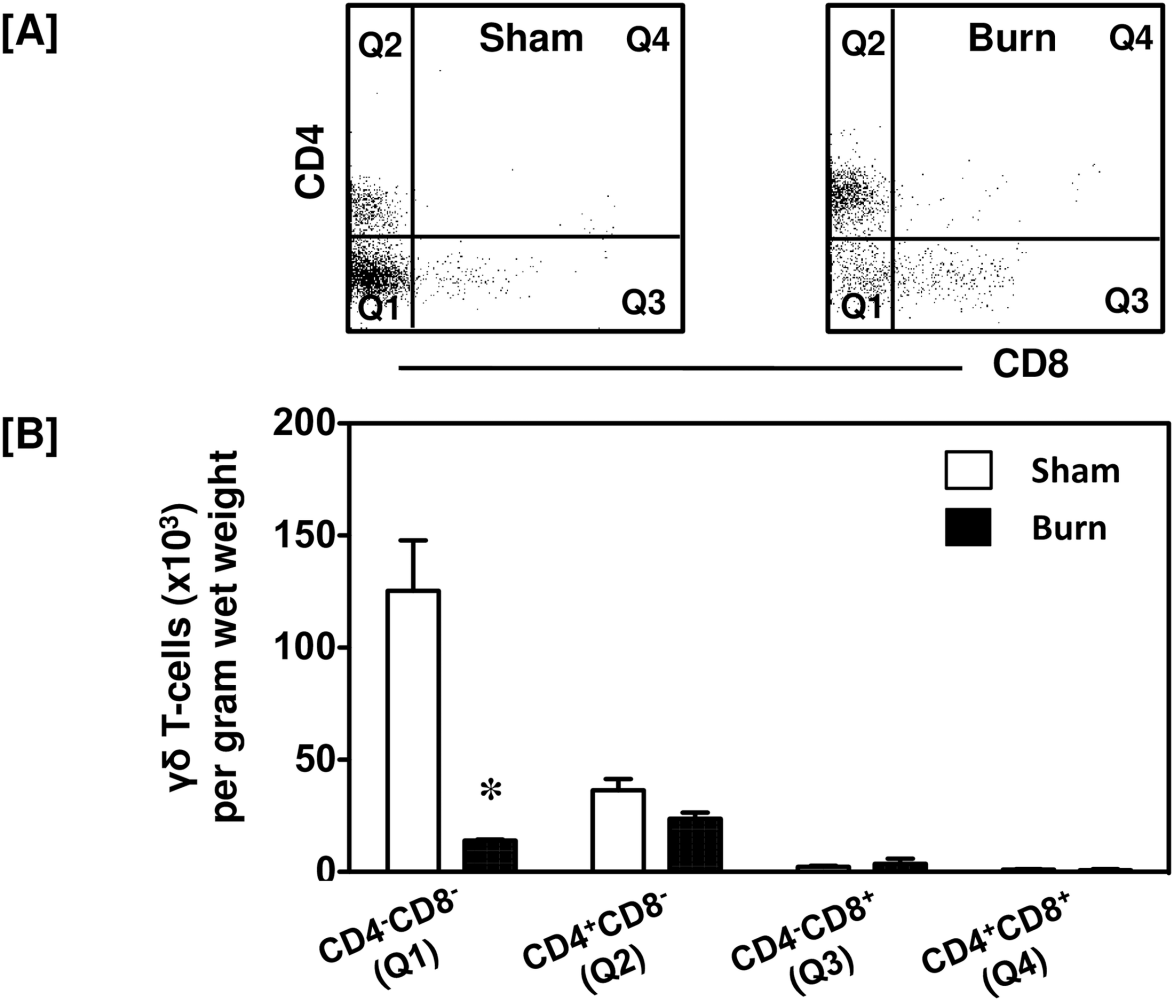
CD4 and CD8 populations of γδ T-cells. After 24 h of sham or burn procedure, skin cells were studied to characterize CD4 and CD8 populations of γδ T-cells using flow cytometry. [A] Gating strategy demonstrating CD4^-^CD8^-^ (Q1), CD4^+^CD8^-^ (Q2), CD4^-^CD8^+^ (Q3) and CD4^+^CD8^+^ (Q4) γδ T-cells from sham and burn mice. [B] Graphical representation of CD4 and CD8 populations of γδ T-cells. Data are mean ± SEM for 5–9 mice/group; *p < 0.05 vs. respective sham.

The T-cells found in sham skin were mainly composed of γδ T-cells and they were decreased significantly in the burn wound (Fig 3). Further characterization of these cells revealed that the vast majority of them in sham skin (76%) were CD4^-^CD8^-^ with 22% being of the CD4^+^CD8^-^ subset (Fig 5A). In the burn wound the CD4^+^CD8^-^ population increased to 59% of the γδ T-cells present, whereas the double negative population decreased to 36%. In comparison to γδ T-cells from sham mice only the actual numbers of CD4^-^CD8^-^ decreased in the burn wound (Fig 5B).

### Burn alters the CD4:CD8 ratio at the wound site

The CD4:CD8 ratio of CD3^+^ T-cells is shown in Table 1. A significant elevation in the numbers of helper (CD4^+^) and (CD8^+^) T-cells was found at the wound site as compared with skin from sham mice. A marked decrease in the CD4:CD8 ratio was observed in the burn wound as compared with uninjured skin from sham mice (0.26 vs. 0.71 for burn and sham, respectively). This change in the CD4:CD8 ratio was predominately due to a greater influx CD8^+^ cells as compared with CD4^+^ T-cells. While the numbers of both T-cell populations increased in the burn wound, CD8^+^ cells increased ~15-fold whereas CD4^+^ cells increased ~50-fold as compared with sham skin levels.

**Table 1:** Cell numbers of CD4 and CD8 subsets.

Total T-cells (x10^3^) per gram wet weight of skin tissue			
	CD4^+^CD8^-^	CD4^-^CD8^+^	CD4:CD8 Ratio
**Sham**	4.8 ± 3.9	62.7 ± 6.4	0.71± 0.01
**Burn**	259.2 ± 110.3*	989.6 ± 361.8*^†^	0.26± 0.07*

Isolated single cells from sham and burn mice were studied for CD4 and CD8 populations of total T-cells using flow cytometry. Data are mean ± SEM for 4 mice/group;

* p < 0.05 vs. respective sham,

^†^ p<0.05 vs. CD4^+^CD8^-^ group.

### CD69 expression on αβ and γδ T-cells is differentially affected by burn

**Fig 6:**
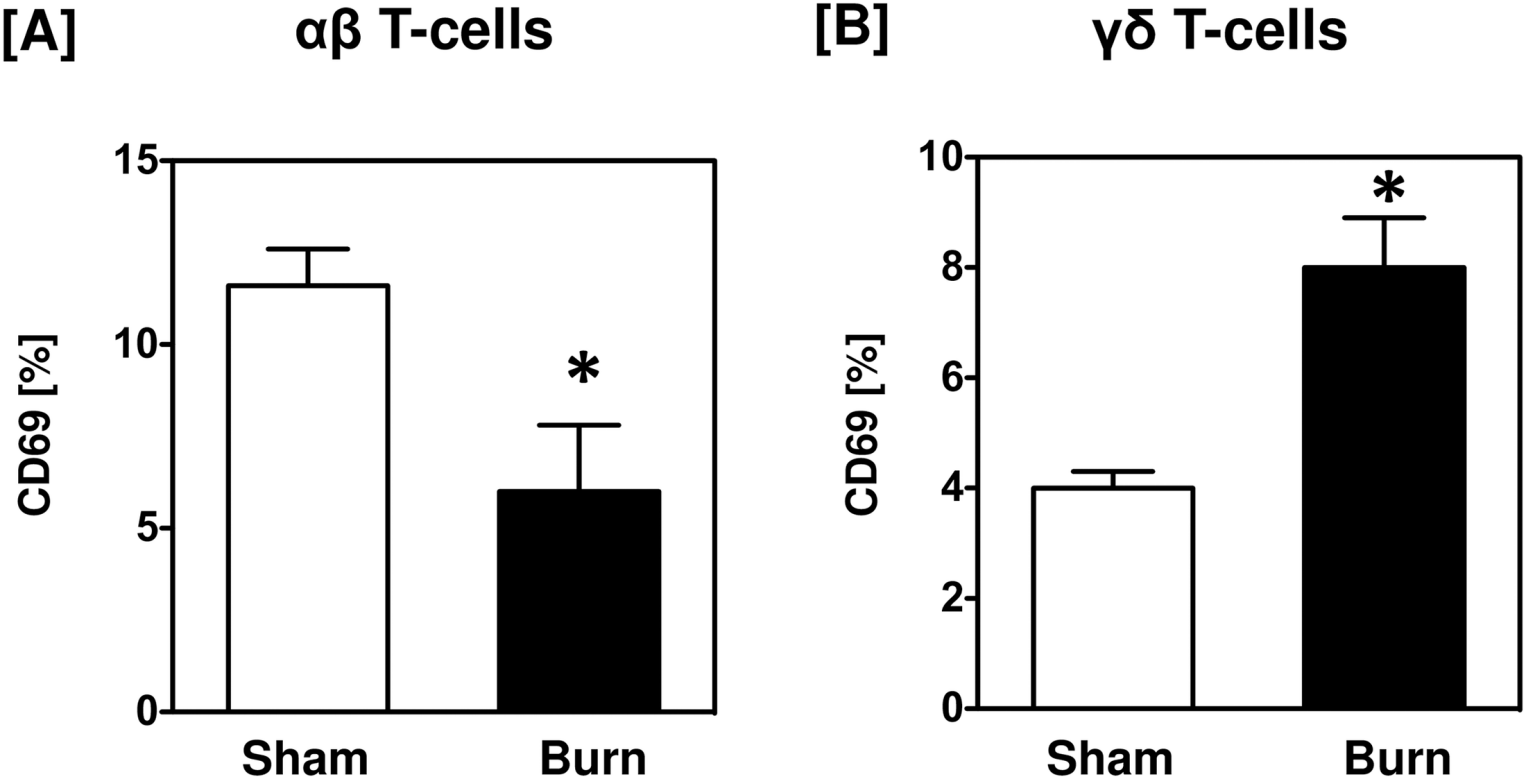
CD69 expression on αβ and γδ T-cells. After 24 h of sham or burn procedure, skin cells were studied for the activation marker, CD69, by αβ and γδ T-cells using flow cytometry. [A-B] Graphical representation of CD69 percentages by αβ and γδ T-cells, respectively. Data are mean ± SEM for 5–9 mice/group; *p < 0.05 vs. respective sham.

Analysis of αβ T-cells for their activation status (CD69 expression) revealed that overall CD69 expression was low and significantly reduced in the burn wound (Fig 6). Similarly, only a small percentage of γδ T-cells expressed CD69; however, in contrast to αβ T-cells, the percent of γδ T-cells expressing CD69 doubled at the wound site as compared with sham skin (Fig 6). In terms of cell numbers (normalized per gram of wet weight of tissue), the number of αβ T-cells in sham skin that were positive for CD69 expression was comparable to that of γδ T-cells (8.6 x 10^3^ vs. 6.2 x 10^3^ cells/g wet tissue). However, after burn, the absolute number of CD69^+^ T-cells decreased overall. With regard to the T-cell subsets, the number of CD69^+^ αβ T-cells was increased significantly at the burn wound when compared to wound CD69^+^ γδ T-cells (4.9 x 10^3^ vs. 1.0 x 10^3^ cells / g wet tissue).

## Discussion

The association between inflammation, involving the complex interaction of various immune cells, and successful wound healing has been well documented [4, 5, 19, 20]. Our laboratory has demonstrated an important role for T-cells in the burn wound inflammatory milieu, recruitment of immune cells to the injury site and healing [8–10, 21]. Based on this central role of T-cells in the burn wound, the current study was performed to further characterize the wound T-cell populations.

Consistent with our previous findings, we observed a marked elevation in the wound T-cell populations [10]. This is consistent with the findings of Purcell et al.[22] whom demonstrated an expansion of the CD4^+^ T-cell population in the draining lymph nodes of the burn site. We found that the CD4:CD8 T-cell ratio was decreased in the burn wound. This decreased T-cell ratio was due to a marked elevation in CD8^+^ T-cells as compared with CD4^+^ cells after burn resulting in a depressed CD4:CD8 T-cell ratio. A low CD4:CD8 ratio has been observed in non-healing venous leg ulcers and increased numbers of CD8^+^ “down-regulatory” suppressor T-cells may have contributed to such complications [23, 24]. Studies suggest that CD4^+^ T-cells promote wound healing, whereas and CD8^+^ T-cells are inhibitory to the process [25, 26].

One of the more striking observations we made was the profound expansion of the “double negative” CD4^-^CD8^-^ αβ T-cell population in the burn wound as compared with normal skin. Early studies from Herndon’s group [27] showed a similar double negative population in the spleens of burn mice. These cells were suppressive as they inhibited lymphocyte proliferation and supported a Th2 type phenotype. Double-negative T cells normally exist as a small (1%–5%) population of lymphocytes in the blood and lymphoid tissues and have been shown to be important in immune regulation, host defense and inflammation [28]. Crispin et al. [29] described an IL-17 producing double-negative T-cell in lupus patients. Interesting Sasaki et al. [18] showed a marked elevation in IL-17 in the burn wound early after injury. It can be speculated that this wound IL-17 spike was a result of the infiltrating double-negative T-cells.

On the other hand, we found that the γδ T-cells from the normal uninjured skin were predominantly of a CD4^-^CD8^-^ double negative phenotype that was decreased significantly in the burn wound. Double negative CD4^-^CD8^-^ T-cells have been shown to control important immunological functions in various human diseases and are known to be inflammatory and regulatory in nature [30, 31]. A high frequency of CD4^-^CD8^-^ αβ T-cells observed in *M*. *tuberculosis*-infected patients was associated with disease severity. The group also demonstrated that CD4^-^CD8^-^ population of αβ and γδ T-cells expressed selective cytokines based on the disease severity. Low disease severity was associated with a pro-inflammatory cytokine profile, whereas severe tuberculosis was associated with enhanced IL-10 production. Based on this data, we may speculate that high frequency of CD4^-^CD8^-^ αβ T-cells present at the wound site may regulate the severity and the outcome of the burn injury and associated wound healing. We speculate that double negative CD4^-^CD8^-^ αβ T-cells accumulate in the burn wound in order to regulate the other immune cells such as CD8^+^ αβ T-cells or CD4^-^CD8^-^ γδ T-cells. Also, CD4^-^CD8^-^ αβ T-cells have been shown to be involved in antiviral and antibacterial defense [32]. Thus, these CD4^-^CD8^-^ αβ T-cells may also be involved in limiting wound site infections.

Burn wound αβ T-cells had significantly reduced CD69 expression, suggesting a down regulation of these cells. In contrast, burn wound γδ T-cells were activated as evidenced by increased CD4 and CD69 expression. There is a growing body of literature supporting this data of activated γδ T-cells after inflammation, injury and/or infection [5, 33, 34].

Our study herein is limited by the lack of a functional characterization of the T-cell subsets, such as cytokine production or myeloid cell activation. Nonetheless, previous work from our laboratory has shown that functionally wound γδ T-cells regulate T-cell and myeloid suppressor cell infiltration, myeloid cells responses (cytokines and iNOS expression) and are important in the regulation of wound growth factor levels [8, 10, 21, 35]. The wound infiltrating αβ T-cells support a Th-2 and Th-17 response that is evident at later times post-injury (i.e., 3–7 days) [10, 35] How the burn-induced changes in the T-cell subsets shown in the current study impact burn wound cell functions and wound healing remains to be determined.

## Conclusions

The results presented herein demonstrate the development of a suppressed T-cell response (i.e., decreased CD4:CD8 ratio) in the burn wound and possible involvement double-negative T-cells in burn wound pathogenesis. The mechanism by which these changes in wound T-cells occurs is likely multifactorial and related in part to burn-induced changes in growth factors, cytokines, and hematopoietic responses [8, 18, 36]. These double-negative T-cells are likely candidates in quelling the early inflammatory response to injury via suppressing lymphocyte proliferation, suppression of myeloid cell activation via Th-2 cytokines and/or recruitment of myeloid suppressor cells. In this regard, we speculate that these T-cells may be important in regulating the transition from the inflammatory to tissue remodeling stages of wound healing. Thus, this unique wound T-cell population may be an important therapeutic target for improving wound healing and recovery in burn patients.
